# Supplementary material for: Effects of water quality, sanitation, handwashing and nutritional interventions on enteric pathogen seroconversion in rural Kenya: a cluster randomised trial
Source: medRxiv. 2026 Nov 1:2026.10.30.26366882. Preprint. [Version 1] doi: 10.64898/2026.10.30.26366882 (PMC13734199; doi:10.64898/2026.10.30.26366882)
Supplement: Supplementary file 1 [file media-1.pdf]

Supplementary material

Effects of water quality, sanitation, handwashing and nutritional interventions on enteric pathogen seroconversion in rural Kenya: a cluster randomised trial

Henry Kanyi*, Richelle W. Kihoro*, Huiyu Hu*, Henry Emisiko, E. Brook Goodhew, Chabier Coleman, Richelle C. Charles, Edward T. Ryan, Claire Munroe, Robert Atmar, Pearl Anne Ante-Testard, Everlyn Kamau, Samantha J. Bents, Beryl S. Achando, Andrew N. Mertens, Holly N. Dentz, Gouthami Rao, Marlene K. Wolfe, Christine P. Stewart, Amy J. Pickering, Clair Null, Audrie Lin, John M. Colford Jr., Patrick J. Lammie, Jeffrey W. Priest, Benjamin F. Arnold†, Sammy M. Njenga†

* Equal contribution, † Equal contribution

Supplementary Figure 1: Luminex IgG response (MFI-bg) to enteric pathogens during visits 1 and 2 in the WASH Benefits Kenya environmental enteric dysfunction substudy. Children were median ages 3 months (visit 1) and 14 months (visit 2) at the time of measurement. Vertical lines mark seropositivity cutoffs derived using external samples (solid) for protozoan and helminth pathogens, and from longitudinal boosting in a pre-exposed cohort approach (dashed).

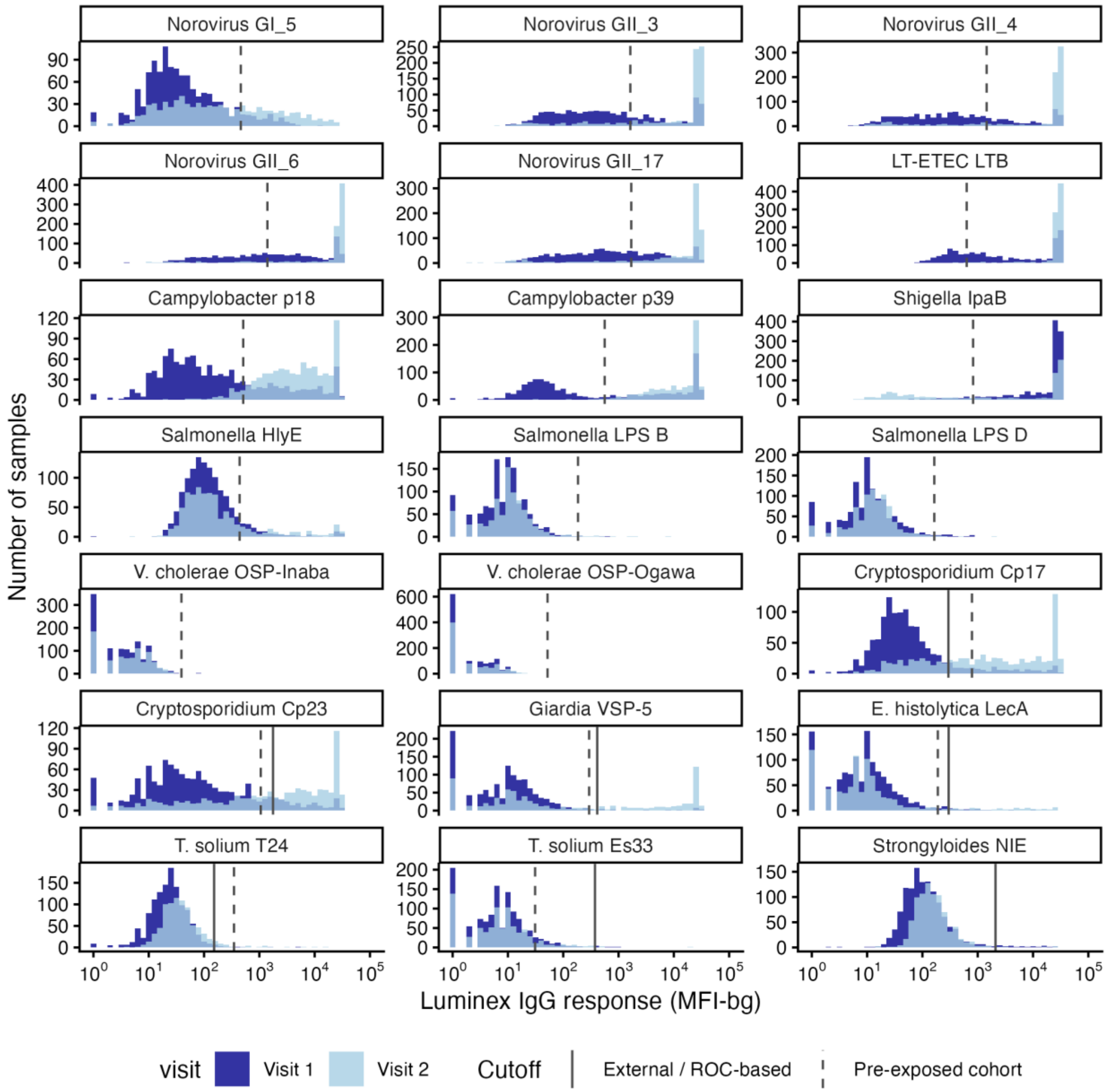

Supplementary Figure 2. CONSORT diagram for the WASH Benefits Kenya trial serology study. DBS, dried blood spot; EED, environmental enteric dysfunction; WASH, water, sanitation, and handwashing.

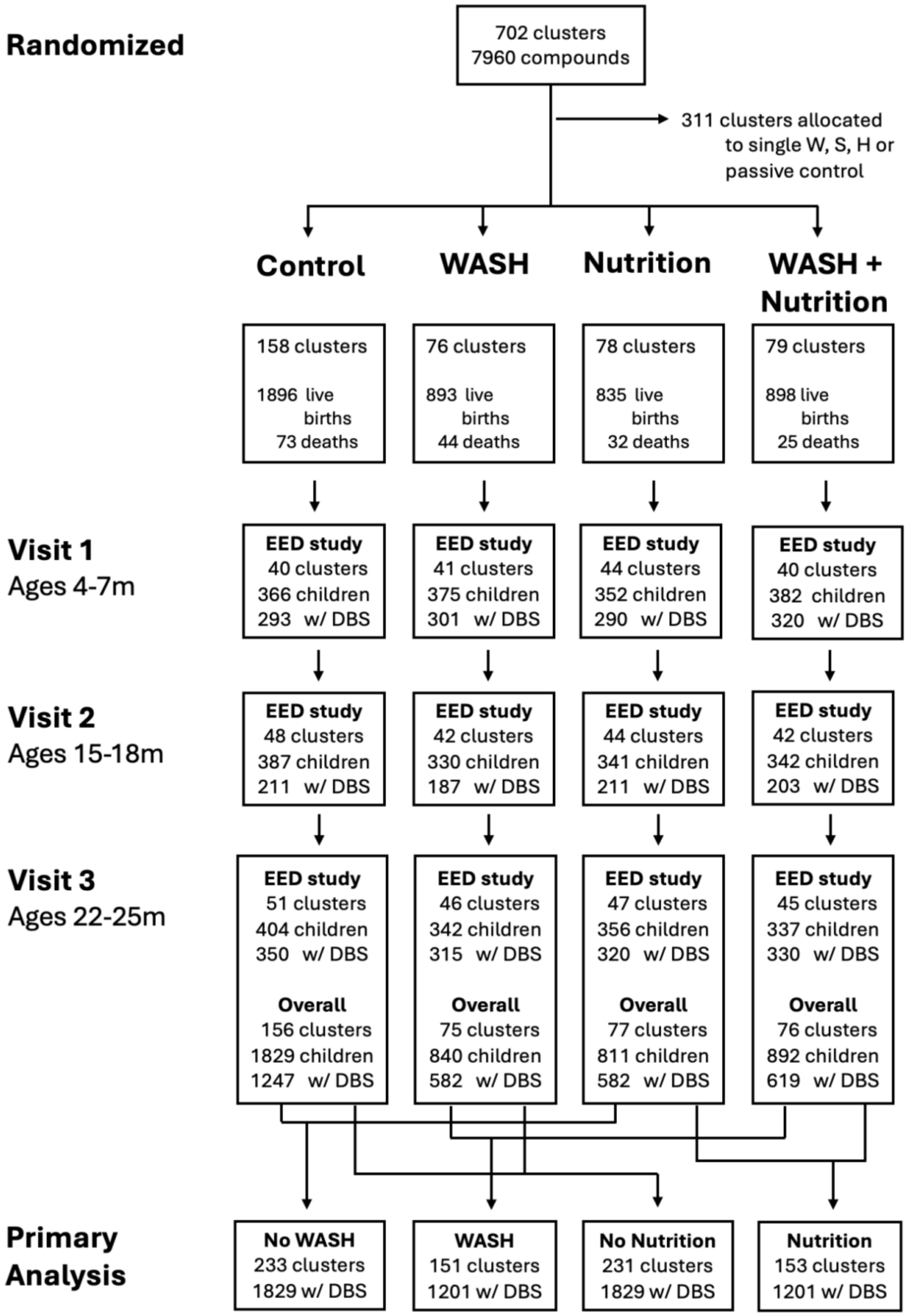

Supplementary Table 3. Baseline characteristics by intervention group in the WASH Benefits Kenya trial. Data are mean (SD) or n (%). Percentages are calculated among participants with nonmissing data for each characteristic.

Characteristic	Control (N=1247)	WASH (N=582)	Nutrition (N=582)	Nutrition + WASH (N=619)
**Maternal**				
Age (years)	26.5 (6.3)	26.6 (6.2)	26.5 (6.2)	26.5 (6.4)
Completed at least primary education	589 (47%)	261 (45%)	275 (47%)	289 (47%)
Height (cm)	160 (5.9)	160 (5.8)	160 (5.8)	160 (6)
Study child is firstborn	232 (19%)	94 (16%)	124 (21%)	122 (20%)
**Paternal**				
Completed at least primary education	707 (61%)	338 (61%)	350 (64%)	348 (60%)
Works in agriculture	526 (44%)	257 (46%)	250 (45%)	270 (45%)
**Household**				
Number of households per compound	1.6 (1.1)	1.7 (1.1)	1.7 (1.2)	1.7 (1.2)
Number of people per compound	8.2 (5.4)	8.3 (5.2)	8.3 (6.1)	8.6 (5.5)
Number of children <18 years in household	2.9 (2)	2.8 (2)	2.9 (2)	3.1 (2.1)
Has electricity	77 (6%)	39 (7%)	46 (8%)	50 (8%)
Has a cement floor	62 (5%)	29 (5%)	29 (5%)	32 (5%)
Owns a mobile phone	1001 (80%)	468 (80%)	479 (82%)	501 (81%)
Owns a motorcycle	128 (10%)	37 (6%)	52 (9%)	52 (8%)
**Drinking water**				
Primary drinking water source is improved*	934 (75%)	408 (70%)	425 (73%)	472 (77%)
One-way walking time to primary water source (min)	10.5 (11.5)	11 (13.3)	11.3 (11.6)	10.7 (12.3)
Reported treating stored water	128 (12%)	65 (14%)	56 (12%)	70 (14%)
**Sanitation**				
Own any latrine	1035 (83%)	494 (85%)	493 (85%)	522 (85%)
Access to improved latrine	200 (17%)	92 (17%)	81 (15%)	83 (14%)
Human faeces observed in the compound	104 (8%)	48 (8%)	57 (10%)	61 (10%)

**Handwashing location**				
Has water within 2 m of handwashing location	312 (25%)	156 (27%)	145 (25%)	167 (27%)
Has soap within 2 m of handwashing location	97 (8%)	80 (14%)	57 (10%)	69 (11%)
**Food security**				
Prevalence of moderate-to-severe household hunger	124 (10%)	63 (11%)	63 (11%)	69 (11%)

Supplementary Table 4. Conditional and marginal seroprevalence by pathogen at 24 month-visit in in the WASH Benefits Kenya trial. Median (IQR) age at the time of measurement was 23 (22, 25) months.

Prevalence by treatment group		Marginal prevalence, combining groups		Risk difference (95% CI)	P value
**Group**	**n/N (%)**	**Group**	**n/N (%)**		
**Viruses**					
**Norovirus GI/GII**					
Control	1171/1247 (93.9%)	No WASH	1715/1829 (93.8%)	Ref	-
WASH	550/582 (94.5%)	WASH	1131/1201 (94.2%)	0.4 (-1.2, 2.1)	0.63
Nutrition	544/582 (93.5%)	No Nutrition	1721/1829 (94.1%)	Ref	-
WASH + Nutrition	581/619 (93.9%)	Nutrition	1125/1201 (93.7%)	-0.8 (-2.4, 0.8)	0.39
**Bacteria**					
***Campylobacter* (p18, p39)**					
Control	1204/1247 (96.6%)	No WASH	1766/1829 (96.6%)	Ref	-
WASH	570/582 (97.9%)	WASH	1164/1201 (96.9%)	0.7 (-0.5, 1.9)	0.33
Nutrition	562/582 (96.6%)	No Nutrition	1774/1829 (97%)	Ref	-
WASH + Nutrition	594/619 (96%)	Nutrition	1156/1201 (96.3%)	-1.1 (-2.3, 0.2)	0.14
***V. cholerae* (OSP-Ogawa, OSP-Inaba)**					
Control	22/1246 (1.8%)	No WASH	27/1828 (1.5%)	Ref	-
WASH	11/582 (1.9%)	WASH	17/1201 (1.4%)	0.3 (-0.5, 1.0)	0.58
Nutrition	5/582 (0.9%)	No Nutrition	33/1828 (1.8%)	Ref	-
WASH + Nutrition	6/619 (1%)	Nutrition	11/1201 (0.9%)	-1.1 (-1.8, -0.3)	0.02

**LT-ETEC (LTB)**					
Control	1243/1246 (99.8%)	No WASH	1823/1828 (99.7%)	Ref	-
WASH	582/582 (100%)	WASH	1200/1201 (99.9%)	0.2 (0.0, 0.5)	0.21
Nutrition	580/582 (99.7%)	No Nutrition	1825/1828 (99.8%)	Ref	-
WASH + Nutrition	618/619 (99.8%)	Nutrition	1198/1201 (99.8%)	-0.1 (-0.4, 0.2)	0.75
**S. enterica typhi (HlyE)**					
Control	280/1246 (22.5%)	No WASH	424/1828 (23.2%)	Ref	-
WASH	132/582 (22.7%)	WASH	291/1201 (24.2%)	0.3 (-2.6, 3.2)	0.88
Nutrition	144/582 (24.7%)	No Nutrition	412/1828 (22.5%)	Ref	-
WASH + Nutrition	159/619 (25.7%)	Nutrition	303/1201 (25.2%)	2.6 (-0.3, 5.5)	0.14
**S. enterica (LPS B,D)**					
Control	88/1246 (7.1%)	No WASH	129/1828 (7.1%)	Ref	-
WASH	36/582 (6.2%)	WASH	88/1201 (7.3%)	0.1 (-1.6, 1.9)	0.92
Nutrition	41/582 (7%)	No Nutrition	124/1828 (6.8%)	Ref	-
WASH + Nutrition	52/619 (8.4%)	Nutrition	93/1201 (7.7%)	0.5 (-1.3, 2.3)	0.63
**Shigella/EIEC (IpaB)**					
Control	1039/1246 (83.4%)	No WASH	1516/1828 (82.9%)	Ref	-
WASH	479/582 (82.3%)	WASH	970/1201 (80.8%)	-2.0 (-4.6, 0.7)	0.21
Nutrition	477/582 (82%)	No Nutrition	1518/1828 (83%)	Ref	-
WASH + Nutrition	491/619 (79.3%)	Nutrition	968/1201 (80.6%)	-2.7 (-5.5, 0.0)	0.08

**Protozoans**					
**Cryptosporidium (Cp17, Cp23)**					
Control	989/1247 (79.3%)	No WASH	1456/1829 (79.6%)	Ref	-
WASH	462/582 (79.4%)	WASH	955/1201 (79.5%)	-0.4 (-3.2, 2.5)	0.82
Nutrition	467/582 (80.2%)	No Nutrition	1451/1829 (79.3%)	Ref	-
WASH + Nutrition	493/619 (79.6%)	Nutrition	960/1201 (79.9%)	0.5 (-2.5, 3.4)	0.77
**Giardia (VSP-5)**					
Control	629/1246 (50.5%)	No WASH	908/1828 (49.7%)	Ref	-
WASH	304/582 (52.2%)	WASH	626/1201 (52.1%)	2.6 (-1.3, 6.5)	0.25
Nutrition	279/582 (47.9%)	No Nutrition	933/1828 (51%)	Ref	-
WASH + Nutrition	322/619 (52%)	Nutrition	601/1201 (50%)	-1.1 (-5.0, 2.7)	0.61
**E. histolytica (LecA)**					
Control	113/1246 (9.1%)	No WASH	183/1828 (10%)	Ref	-
WASH	75/582 (12.9%)	WASH	131/1201 (10.9%)	0.4 (-1.9, 2.8)	0.75
Nutrition	70/582 (12%)	No Nutrition	188/1828 (10.3%)	Ref	-
WASH + Nutrition	56/619 (9%)	Nutrition	126/1201 (10.5%)	0.4 (-1.9, 2.8)	0.75
**Helminths**					
**T. solium (Es33, T24)**					
Control	149/1246 (12%)	No WASH	215/1828 (11.8%)	Ref	-
WASH	53/582 (9.1%)	WASH	129/1201 (10.7%)	-1.4 (-3.7, 0.9)	0.30
Nutrition	66/582 (11.3%)	No Nutrition	202/1828 (11.1%)	Ref	-

WASH + Nutrition	76/619 (12.3%)	Nutrition	142/1201 (11.8%)	0.6 (-1.7, 2.9)	0.66
**S. stercoralis (NIE)**					
Control	13/1246 (1%)	No WASH	18/1828 (1%)	Ref	-
WASH	6/582 (1%)	WASH	12/1201 (1%)	0.0 (-0.6, 0.7)	0.96
Nutrition	5/582 (0.9%)	No Nutrition	19/1828 (1%)	Ref	-
WASH + Nutrition	6/619 (1%)	Nutrition	11/1201 (0.9%)	-0.1 (-0.8, 0.5)	0.69

Supplementary Figure 5. Age-dependent seroprevalence for enteric pathogens by nutrition treatment in the WASH Benefits Kenya trial. A. Higher transmission pathogens. **B.** Lower transmission pathogens. The only difference between panels is the Y-axis range, to better show the range of the data. Lines age-seroprevalence estimated by treatment group using locally weighted regression over individual data (points), trimmed to ages 4-28 months to avoid edge effects. Points and error bars to the right of each panel show treatment-specific marginal average seroprevalence and 95% confidence intervals by study visit (1, 2, 3). Gray shading shows child age ranges of visits 1 and 3.

A

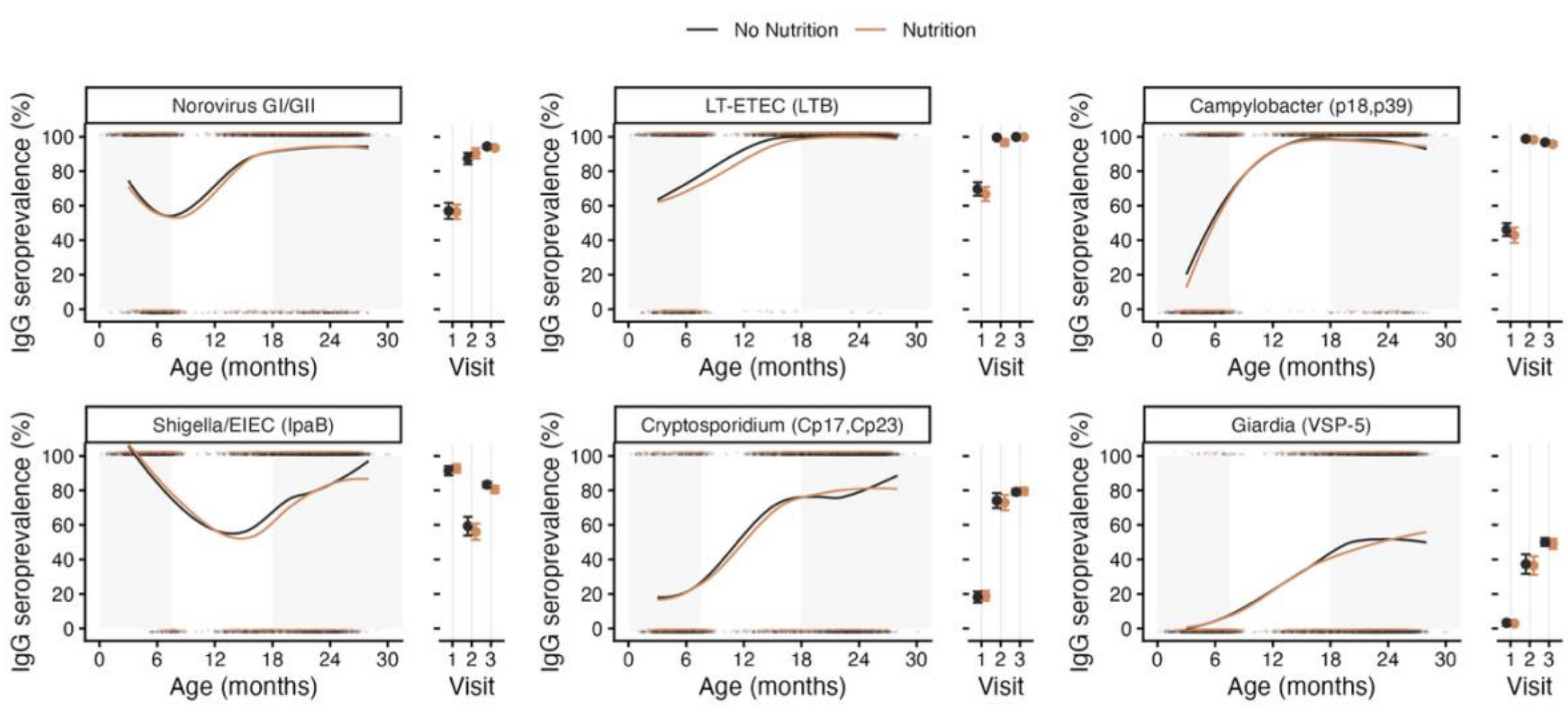

B

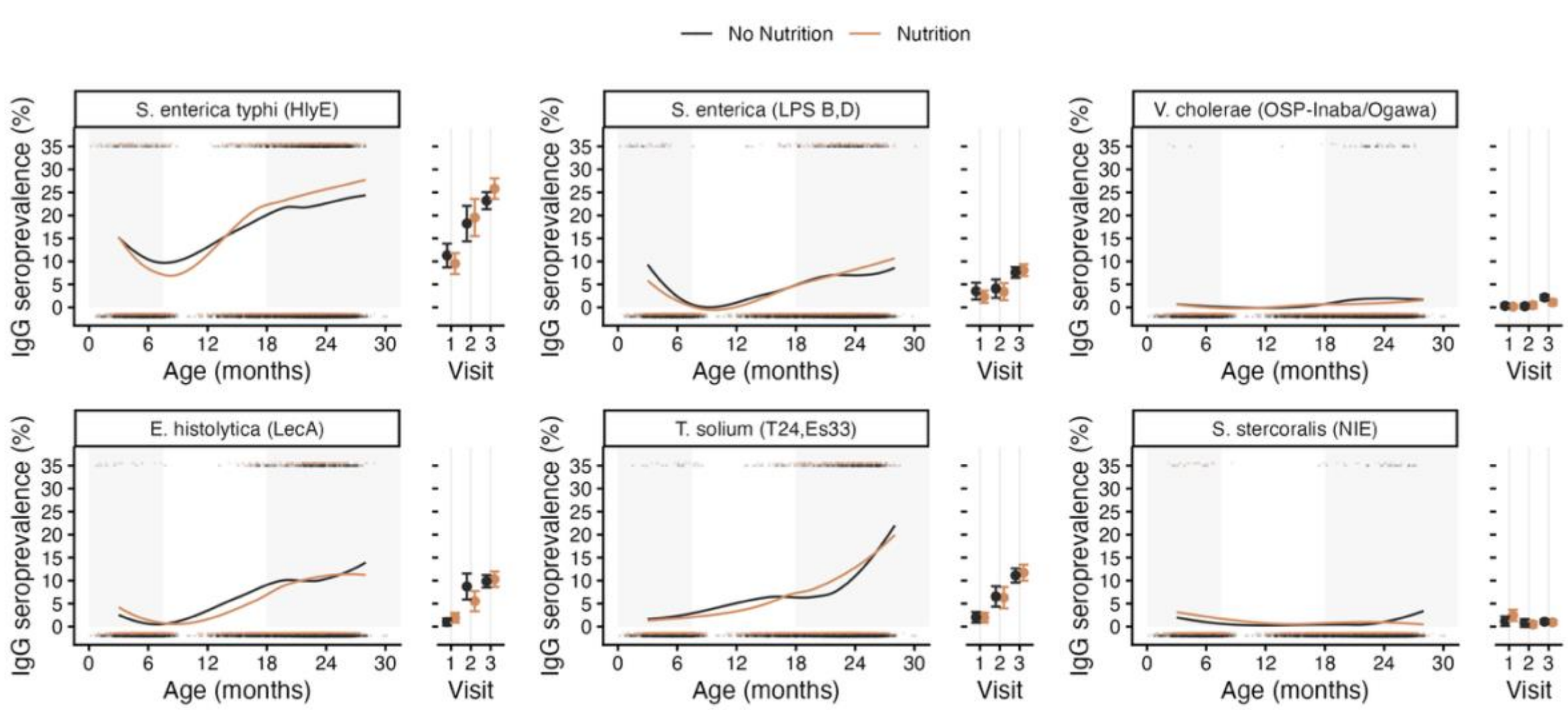

Supplementary Figure 6. Distribution of the number of enteric pathogens to which children were seropositive at the 24-month visit, by WASH and nutrition intervention assignment in the WASH Benefits Kenya trial.

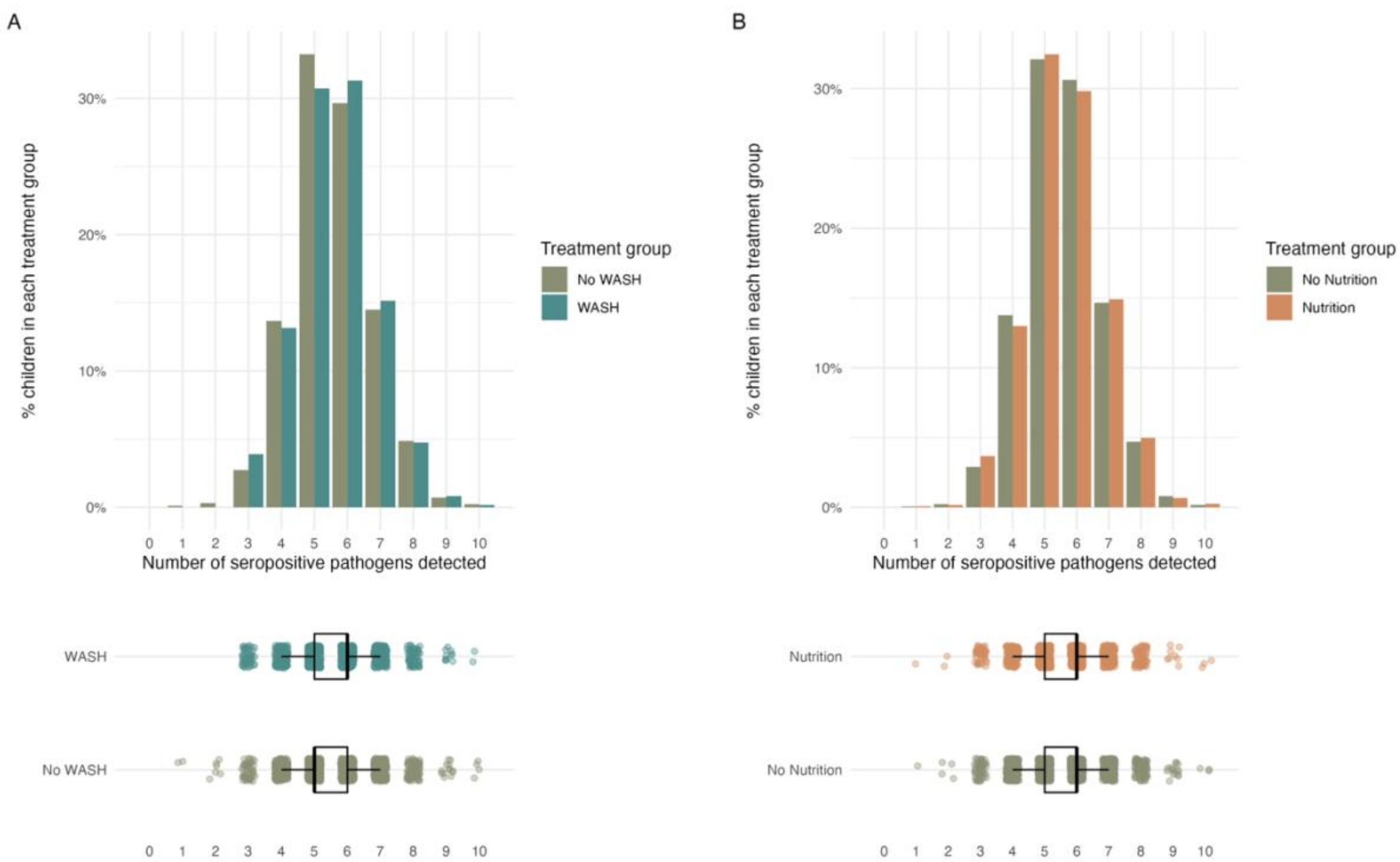

Supplementary Table 7. Intervention adherence at enrollment and at 1- and 2-year follow-up by 2 x 2 factorial WASH and Nutrition margins in the WASH Benefits Kenya trial.

Indicator	Round	WASH factorial margin		Nutrition factorial margin	
		WASH	No WASH	Nutrition	No Nutrition
Number of compounds assessed	Enrollment	1830/1833 (100%)	2756/2762 (100%)	1761/1764 (100%)	2825/2831 (100%)
	Year 1	1029/1833 (56%)	1497/2762 (54%)	947/1764 (54%)	1579/2831 (56%)
	Year 2	1410/1833 (77%)	2108/2762 (76%)	1385/1764 (79%)	2133/2831 (75%)
Visited by promoter in past month	Enrollment	..	..	..	..
	Year 1	774/986 (78%)	1010/1413 (71%)	732/907 (81%)	1052/1492 (71%)
	Year 2	500/1359 (37%)	743/2047 (36%)	510/1345 (38%)	733/2061 (36%)
Stored drinking water has detectable free chlorine	Enrollment	55/1440 (4%)	58/2190 (3%)	40/1390 (3%)	73/2240 (3%)
	Year 1	336/791 (42%)	34/1239 (3%)	165/759 (22%)	205/1271 (16%)
	Year 2	240/1250 (19%)	53/1979 (3%)	143/1266 (11%)	150/1963 (8%)
Access to improved latrine	Enrollment	296/1739 (17%)	428/2582 (17%)	262/1666 (16%)	462/2655 (17%)
	Year 1	897/1003 (89%)	241/1417 (17%)	488/901 (54%)	650/1519 (43%)
	Year 2	1090/1350 (81%)	370/1994 (19%)	660/1319 (50%)	800/2025 (40%)
Child faeces safely disposed of	Enrollment	121/722 (17%)	147/1031 (14%)	89/663 (13%)	179/1090 (16%)
	Year 1	613/895 (68%)	493/1294 (38%)	442/823 (54%)	664/1366 (49%)
	Year 2	424/1254 (34%)	188/1898 (10%)	271/1235 (22%)	341/1917 (18%)
Handwashing location has water and soap	Enrollment	117/1830 (6%)	153/2756 (6%)	110/1761 (6%)	160/2825 (6%)
	Year 1	797/1029 (77%)	185/1497 (12%)	442/947 (47%)	540/1579 (34%)
	Year 2	282/1410 (20%)	203/2108 (10%)	228/1385 (16%)	257/2133 (12%)
LNS sachets consumed (% expected)*	Enrollment	-	-	-	-
	Year 1	96%	95%	95%	-
	Year 2	116%	114%	115%	-

Note. Data are n/N (%) or %. “-” indicates that a measure was not collected or was not interpretable for that factorial margin. Free chlorine was measured in every trial arm. *LNS consumption was reported for the Nutrition group only and is conditional on a child being in a Nutrition cluster, expressed as a percentage of expected consumption. Promoter visits and LNS consumption were not measured at enrollment. WASH = water, sanitation, and handwashing; LNS = lipid-based nutrient supplement.
